# Importance of Surfactant Quantity and Quality on Growth Regime of Iron Oxide Nanoparticles

**DOI:** 10.3390/ma13071747

**Published:** 2020-04-09

**Authors:** Urszula Klekotka, Dariusz Satuła, Anna Basa, Beata Kalska-Szostko

**Affiliations:** 1 Faculty of Chemistry, University of Białystok, Ciołkowskiego 1K, 15-245 Białystok, Poland; u.klekotka@uwb.edu.pl (U.K.); abasa@uwb.edu.pl (A.B.); 2 Faculty of Physics, University of Białystok, Ciołkowskiego 1L, 15-245 Białystok, Poland; d.satula@uwb.edu.pl

**Keywords:** iron oxide nanoparticles, magnetite nanoparticles, surfactant surface modification, magnetic properties, structural characterization, Mössbauer spectroscopy

## Abstract

This study shows the influence of selected nonstandard surfactants on the growth and properties of magnetite nanoparticles. Particles were obtained using thermally decomposed iron (III) acetylacetonate in an organic environment. For synthesis, three different concentrations (4, 8, and 16 mmol) of tested surfactants were used. Five types of each long-chain carboxylic acid and amines were selected for stabilization of nanoparticles. Nanoparticles were characterized by X-ray diffraction, transmission electron microscopy, and infrared spectroscopy. Magnetic properties of the nanoparticles were tested by conventional room temperature Mössbauer spectroscopy with and without external magnetic field. TEM images clearly showed that application of tertiary amines causes the nanoparticles to form nanoflowers, in contrast to other compounds, which do not show such growth. Influence of surfactant amount on growth regime depends on the nature of the substances. Mössbauer spectroscopy confirms differences in magnetic core composition as a result of the surfactant amount present in synthetic procedure.

## 1. Introduction

Nowadays, surface stabilizers are widely used in different areas of human life. They are present in detergents, emulsifiers, foaming agents, as well as in antibiotics or herbicides. They possess the ability of adsorption, and thus can change the surface of the objects on which they are present [1,2].

Due to their special properties, some compounds can be used as substrates in nanoparticle synthesis, where they play the role of surface stabilizers, oxidation protecting agents, and separators in the aggregation process (in magnetic material cases) [3,4]. On the other hand, properly distributed particles can be easily manipulated by an external magnetic field [5]. In addition, when the size of particles decreases below submicron scale, they gain new extraordinary properties that are not typical of reference bulk materials, such as optical, electric, magnetic, and thermodynamic stability, etc. [6,7]. Great surface to volume ratio influences their abnormal chemical activity, and the surface termination establishes final properties which reflect in its application [8]. The existence of a large variety of nanostructures and possible surface stabilizers causes ongoing need for more and more detailed studies. Since the growing regime is determined by competition between surface and core precursors, the same growth scenario regimes cannot be observed even for similar “chemical” substances [9]. Our main goal is to find the best controlled growth of magnetite nanoparticles from selected compounds and recognize process dependence. 

Generally, iron oxide nanoparticles are now widely used in IT memory media, environmental protection, food products, medicine, etc. [10,11,12,13]. They can also be applied for targeted drug delivery in vivo, heating centers, contrast agents, biosensors, as well as adsorption centers of heavy metals in detectors of pollutants [14,15,16,17,18]. Their biocompatibility and antibacterial properties allow for usage in a wide range of clinical tests. However, their surface has to have specific characteristics to be useful. Magnetic nanoparticles can be also applied in other areas of human activity, such as electronics, data carriers, in industry, for production of antibacterial self-cleaning surfaces, or environment protection as water cleaners or catalysts [19,20,21,22]. Due to their wide range of possible application, magnetic nanoparticles are continuously heavily studied in order to obtain increased efficient materials (what can be realized via small size and narrow size distribution, proper chemical activity, and stability in various environments) [23]. Therefore, detailed studies of the composition and their relation on final useful properties are still needed. Literature searches show that with adjustment of the reaction conditions (time and temperature of synthesis, type of surfactant, and precursor concentrations), control of nanocrystal size and growth regime can be achieved [24,25]. For example, a high surfactant/precursor ratio favors the creation of a large number of small nanocrystals in comparison to existing particle growth. In addition, nanoparticles size can be controlled by binding different kinds of surfactants to the growing grains surface. As a result, short chain stabilizers allow for faster growth, which result in bigger particles, contrary to long chain compounds which favors a slower rate [26]. There is also a growing interest in the application of nanoparticles in magnetic hyperthermia. In this area not only core composition and size is important but also its surface termination which governs distance between heating centers [27,28,29]. Huge attention has been paid as well to tumor treatment via thermally stimulated drug delivery based on various types of functionalized magnetic particles [30,31]. In summary, any biorelated application needs well characterized materials where its description includes both fundamental knowledge and usage related tests.

Interplay between the ratio of inorganic core precursors and organic surfactants can cause a few growth regimes, which form a well-specified size of nanoparticles compared to a scatter mixture. Our previous studies suggest that there is a clear dependence of selected surfactant on growth regime [3]. The present study describes the influence of the amount of used substrates on size and defines the properties of nanoparticles.

## 2. Experimental

### 2.1. Material and Apparatus

In order to obtain magnetite nanoparticles (MNPs) with various surface stabilizers types and thicknesses, the following substrates were purchased from Sigma-Aldrich: Fe(acac)_3_, 1,2-hexadecanediol, phenyl ether, oleyl amine, lauric acid, palmitic acid, stearic acid, caprylic acid, trioctylamine, hexylamine, dioctylamine, and triethylamine. Acetone and oleic acid were purchased from Polish Chemical Compounds (POCH). The purification process and subsequent separation of nanoparticles from unreacted residuals were performed by simultaneous usage of acetone, sonication bath, and permanent magnet [32]. 

Crystallinity of all synthesized nanoparticles were checked by X-ray diffraction (XRD) (Agilent Technologies SuperNova diffractometer with a Mo microfocused source (K_α2_ = 0.0713067 nm)). Diameter, shape, and size distribution were estimated based on transmission electron microscopy (TEM) (FEI Tecnai G2 X-TWIN 200 kV microscope, Hilsboro, OR, USA). Verification of surface modification was upon infrared (IR) spectrometry (Nicolet 6700 spectrometer working in reflecting mode, Thermo Fischer Scientific, Waltham, MA, USA). Mössbauer spectra of all samples were obtained with the use of the spectrometer working in constant acceleration mode with a ^57^CoRh radioactive source. For each experiment, a spectrum of metallic iron foil (α-Fe) was used as a reference. All samples were measured at room temperature and in the transmission mode. A hand-made permanent magnet setup was used as a source of homogenous 1.3 T external magnetic field in the sample position (for in field Mössbauer spectroscopy).

### 2.2. Magnetite Nanoparticles Preparation Routine

**Figure 1:**
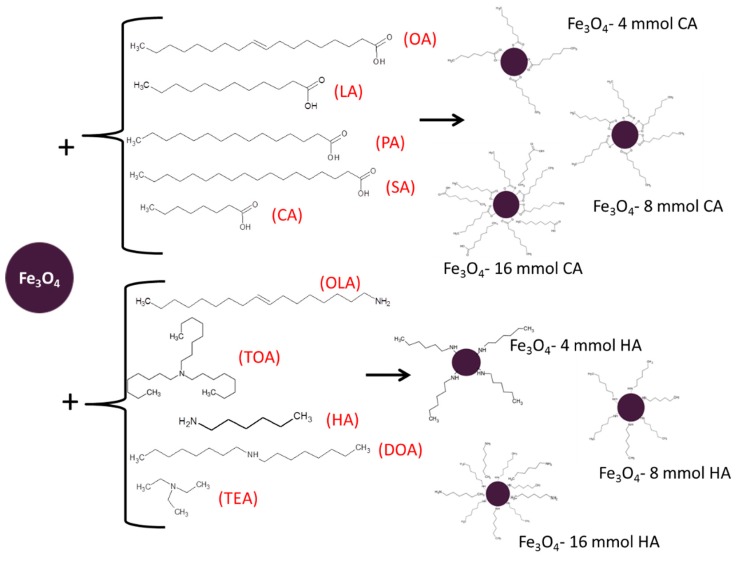
Schematic presentation of the coating by fatty acids and amines.

Studied in the paper magnetite (Fe_3_O_4_), nanoparticles were obtained using a specific routine. First, chemicals Fe(acac)_3_, 1,2-hexadecanediol, and phenyl ether were mixed together in the same amounts for all trials. Then, one of three concentrations (4, 8, or 16 mmol) of one of the different surface stabilizers (oleic acid, lauric acid, palmitic acid, stearic acid, caprylic acid, trioctylamine, hexylamine, dioctylamine, triethylamine, or oleyl amine) were added, respectively. Throughout the whole synthesis, an inert atmosphere was maintained by continuous argon flow. The temperature of the synthesis was kept around 260 °C for 30 min to preserve boiling temperature of phenyl ether [33,34]. Afterwards, the nanoparticles solution was rinsed with deoxygenated acetone and dried in vacuum conditions with use of an evaporator until the powder was obtained. In Table 1, the summary and notation of prepared nanoparticles are displayed. The scheme of synthesis is presented in Figure 1. 

## 3. Results and Discussion

### 3.1. Transmission Electron Microscopy

**Figure 2:**
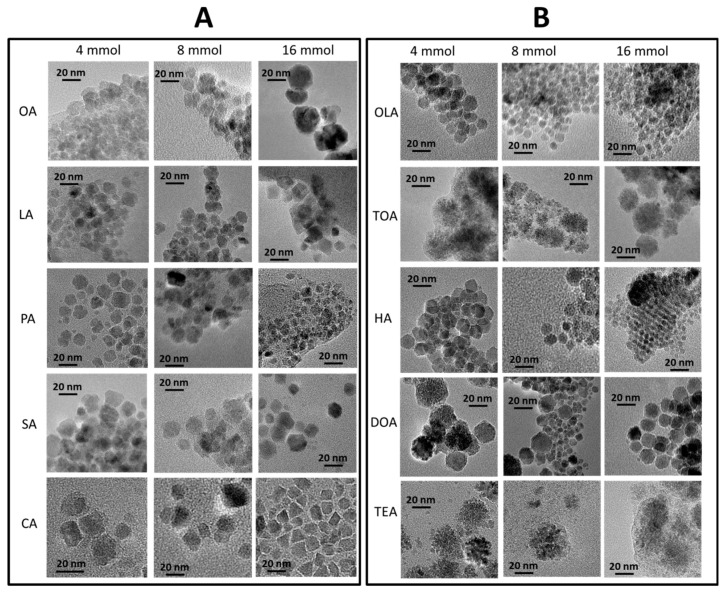
TEM images of synthesized nanoparticles. In the first series (**A**), nanoparticles with only organic acids are presented, in three concentrations separated by each column, in the second series (**B**), with amines and variable concentrations in columns.

Qualitative and quantitative analysis of nanoparticles morphology (size, shape, and size distribution) was done based on transmission electron microscopy. TEM images were collected in series and can be found in Figure 2 (carboxylic acids—panel (A), and amines—panel (B)).

Detailed analysis of TEM images (Figure 2) conclude that the application of different surfactants in various concentrations can determine morphology, diameter, shape, and size distribution of the nanoparticles. In general, usage of amines results in a more even shape of nanostructures. Furthermore, with an increase in concentration of surfactant, there was a decrease in average particle size. Some of the obtained nanoparticles strongly aggregate (Fe_3_O_4_ + 4 mmol of OA, Fe_3_O_4_ + 16 mmol of TEA) in indistinct shapes. The most regular, homogenous, and well-separated nanoparticles were obtained for Fe_3_O_4_ + 4 mmol of OLA, Fe_3_O_4_ + 4 mmol of HA, Fe_3_O_4_ + 16 mmol of HA, Fe_3_O_4_ + 4 mmol of DOA, and Fe_3_O_4_ + 16 mmol of DOA. In a few cases, self-organization ability can be observed, which is only present for well-defined nanoparticles with narrow size distribution [35]. From obtained images, it can be also observed that in the case of tertiary amines (trioctylamine, and triethylamine), particles grow as nanoflowers, regardless of the amount of used surfactant. 

Nanoparticles obtained in the presence of organic acids have wider size distribution and are less regular in shape in comparison to nanoparticles synthesized with amines. Here, a clear conclusion cannot be made because growth changes case by case. The size of particles increases with concentration of LA. On the other hand, presence of CA causes growth of rectangular or triangular particles. Quantitative analysis of the nanoparticles gives the sizes of each type of nanoparticle. Obtained values for each series are presented in Table 2.

### 3.2. X-ray Diffraction

**Figure 3:**
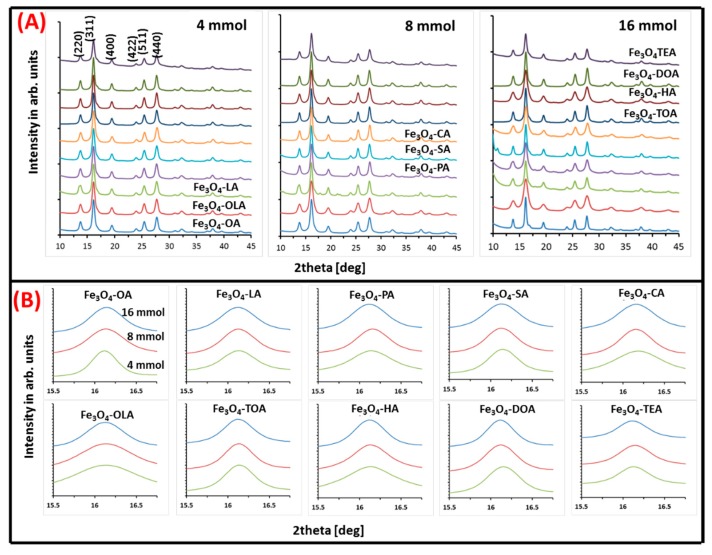
(**A**) Three series of X-ray diffraction patterns of obtained nanoparticles. Each series shows nanoparticles with specific concentrations of tested surfactants; (**B**) zoom on (311) peak in respect to surfactant and its concentration.

With the use of a micro-focused X-ray diffractometer, the crystal structure and concentration of resultant iron oxide nanoparticles grown in the presence of different surfactants was measured. Obtained XRD patterns in series with restriction of concentration are depicted in Figure 3, along with a summary of calculated average grains size, lattice constants, and strains (Table 2). 

All diffractograms presented in Figure 3A show typical inverse spinel set of patterns indicating growth of magnetite/maghemite structure. According to Miller nomenclature, present peaks can be indexed as (220), (311), (400), (422), (511), and (440) [36]. The position on the 2θ angles axis and relative intensity of observed signals proves presence of magnetite/maghemite phases. No other iron oxides are observed, which justify universality of the chosen fabrication procedure. 

Zoom on (311) (Figure 3B) peak allows to correlate TEM images with quality and eventual transition of the particle’s crystal structure, which is reflected as a small shift of maximum of the peak on 2θ axis and changes of its linewidth.

Obtained values for each type of particle (lattice constant, strain, and grain size) are presented in Table 2. Lattice constant values vary between (8.35–8.40 ± 0.02) (Å), which is in agreement with the literature value of bulk magnetite and maghemite lattice constants (8.39 Å) [37], (8.33 Å) [38], respectively. However, in most cases, obtained numbers are closer to the magnetite value. Therefore, it can be concluded that the amount of maghemite phase is very small, and probably present only at the nanoparticles surface. Nanoparticle grain size and strain, which can indicate crystal imperfections, were calculated with the use of Williamson–Hall Equation (1) [39]:

(1) $$\beta \cos ϴ=\left(\frac{0.9\lambda }{D}\right)+\left(4\varepsilon \sin ϴ\right)$$

where β—full width at half maximum in (rad); ϴ—diffraction angle in (rad); λ—wavelength in (Å); D—grain size in (Å); ε—strain (dimensionless).

From the obtained values (Table 2), it can be seen that the grain size of the nanoparticles strongly depends on the type and amount of used surface stabilizers. Therefore, not only is the type of surfactant important in the synthesis environment, but also the amount of organic ingredients used in the procedure is vital in the production of nanoparticles. Both factors influence the nanoparticles growth process, however their relative relation cannot be summarized in a general rule. Strain decreases with larger amounts of surfactant, but not in all cases (exclude LA, SA, TOA, TEA). Values of average grain size vary between (6–14 ± 2) nm. It is observed that, in the case of amines crystallites, diameter clearly decreases (oleylamine, hexylamine, dioctylamine) with concentration change, which is in line with TEM observation. Some values disagree with the TEM data, nevertheless general trends are well presented. The biggest disagreement is seen in TOA and TEA data. The average diffracting zone is not well correlated with particles size due to its internal structures. Such discrepancy can be accepted because comparison considers results of two methods in which sampling and results fitting is different. As a consequence, therefore, the amount of specified material is not the same for both and it is a source of inconsistency in obtained values. It should be noted that the fitted in procedure values are affected by uncertainty connected with maghemite and magnetite crystal parameters, composition distribution, crystal surface distortion, as well as greater influence of strain in smaller particles [40]. Strain values for all samples were evaluated, as well. The smallest particles and the one with irregular morphology show strain values higher than 3.5 × 10^−3^.

### 3.3. IR Spectroscopy

**Figure 4:**
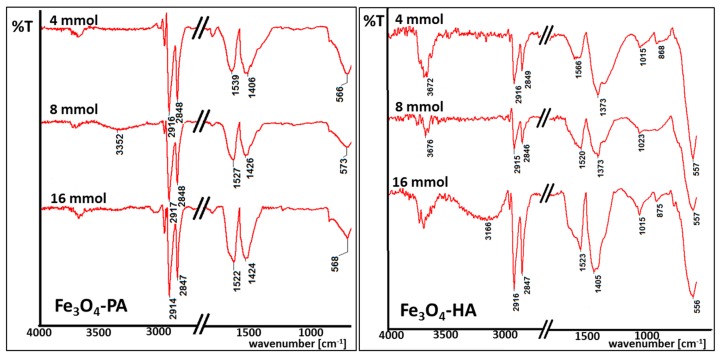
IR spectra of magnetic nanoparticles coated with palmitic acid (Fe_3_O_4_-PA) and hexylamine (Fe_3_O_4_-HA) in tested concentrations.

Magnetic nanoparticles obtained in performed studies were analyzed by IR spectroscopy. IR spectroscopy was required to detect induced changes on the nanoparticles surface caused by the usage of diverse surfactants. Obtained spectra were collected for all samples, but due to similarity between spectra, only one set from each kind (acid or amine) are presented in this paper (Figure 4). The number of specific bands for nanoparticles coated with palmitic acid or hexylamine were clearly found which proved successful surface functionalization. For clarity, the middle part of the spectra was omitted. 

Selected IR spectra depicted in Figure 4 are typical for iron oxide nanoparticles coated with long-chain surfactants. Wide bands observed at 3500 cm^−1^ are typical for O-H bonds, which can be present due to adsorbed vapor water. Signals at 2900–2800 cm^−1^ are connected with the presence of C-H bonds in carbon chains of all tested organic compounds. Strong signals in the spectral range 1420–1530 cm^−1^ are typical for COO^−^ bonds present in the acetylacetonate groups (iron oxide nanoparticles precursor). In terms of amines surfactants, signals at 1015–1020 cm^−1^ can be seen, which illustrate the presence of N-H bonds at the nanoparticles surface [41]. Every spectra shows intense signals at around 580 cm^−1^, which clearly proves the presence of Fe-O bonds in magnetite [42,43]. In most cases, a proper ratio of Fe-O signals is observed to these typical for the surfactant carbon chain. It changes with the amount of used surfactant or/and particle size.

### 3.4. Mössbauer Spectroscopy

**Figure 5:**
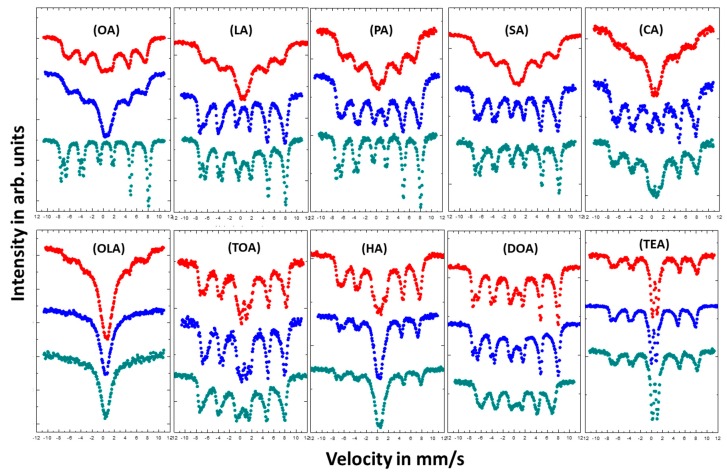
Room temperature (RT) Mössbauer spectra of magnetite nanoparticles with different coatings. Color of the spectra determines the amount of added surfactant: Red—4 mmol; blue—8 mmol; and green—16 mmol.

**Figure 6:**
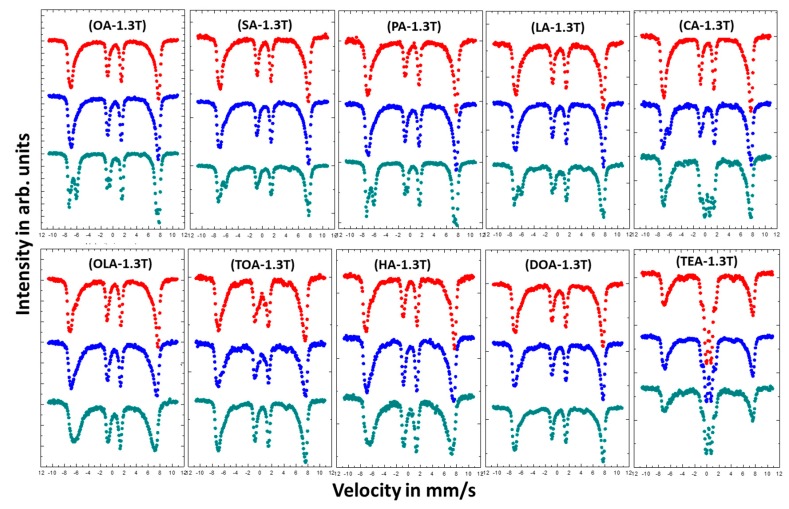
Mössbauer spectra of magnetic nanoparticles with different coatings measured in external magnetic field of 1.3 T parallel to gamma beam direction. Colors of the spectra determines the amount of added surfactant: Red—4 mmol; blue—8 mmol; and green—16 mmol.

Studies on the effect of coating on magnetic properties of magnetite nanoparticles were conducted by room temperature (RT) Mössbauer spectroscopy. Figure 5 shows measured spectra, which are depicted in a series depending on used surfactant and its concentration. The Mössbauer spectra present in Figure 6 were measured at RT in an external magnetic field of 1.3 T parallel to gamma beam direction.

As seen by collected Mössbauer spectra presented in Figure 5, magnetic response of each type of nanoparticle varies dependent on nanoparticle size and surfactant concentration, which is directly correlated to particle–particle interaction via surfactant layers. Surface stabilizers change growth regime in relation to its concentration in reacting volume. In addition, stabilizers can prevent the oxidation process during preparation of the nanoparticles. The shapes of the spectra measured for nanoparticles coated with OA, LA, PA, SA, and CA change from a broad sextet typical for superparamagnetic fluctuation of Fe magnetic moments for low concentration of the surfactant (4 or 8 mmol), to the spectra typical for bulk magnetite which can be described as superposition of the two sharp subspectra with distinguished hyperfine parameters (for 16 mmol). This comes from the Fe atoms located in the [A] and [B] position in the magnetite invers spinel structure [44]. Such conversion of the spectral shapes means that the increase of surfactant concentration led to the suppression of the superparamagnetic fluctuation of the Fe magnetic moments. For OLA, TOA, HA, DOA, and TEA the influence of surfactant concentration on the shape of the spectra is rather small. It was expected that the increase of the surfactant concentration should increase the superparamagnetic fluctuation because of the weakening dipole–dipole interaction between nanoparticles. That is related to the rise of the distance separating objects. Such scenario is also preventing the agglomeration process, which easily appears among magnetic grains. Our observations have shown the contrary.

In order to obtain more information on the magnetic arrangement of Fe magnetic moments, measurements in an external magnetic field were carried out (Figure 6).

Almost all measured in external magnetic field Mössbauer spectra (except TEA) do not show any superparamagnetic fluctuation of the Fe magnetic moments at RT. This means that external magnetic field of 1.3 T is enough to suppress superparamagnetic behavior in studied samples. Moreover, the intensities of the 2nd and 5th line in the spectra are reduced to almost zero. The reason for this is that the studied nanoparticles are magnetically very soft and the magnetic moments are easily arranged parallel to external magnetic field direction. Such reaction of magnetic moments on external magnetic field are typical for magnetite and/or maghemite nanoparticles in contrast to hematite nanoparticles, where magnetic moments tend to arrange perpendicular to external magnetic field [45]. Since the measurements in an external magnetic field cause suppression of superparamagnetic fluctuation of the Fe magnetic moments, the spectra become less complicated and have better resolution. Therefore, the shapes of the spectra can be distinguished as magnetite (two separate sextets) or maghemite (two combined sextets). The obtained results for nanoparticles coated with OA, LA, PA, SA, and CA show that the highest concentration (16 mmol) of the surfactant ensure growth of magnetite nanoparticles, while concentration of 4 and 8 mmol lead to maghemite or so called nonstoichiometric magnetite [46]. This means that the increase of surfactant concentration prevents the surface oxidation process during the preparation procedure. The scenario of the variable nanoparticles growth regime as a function of surfactant concentration can explain the suppression of superparamagnetic fluctuation for higher surfactant quantity. Magnetite nanoparticles are observed as being more stable with regard to superparamagnetic fluctuations than maghemite [47], which gives them advantage in eventual magnetic hyperthermia applications [48]. In the case of OLA, TOA, HA, DOA, and TEA such effect is not visible. The increasing of stabilizers concentration does not influence much on the shapes of measured spectra. 

## 4. Conclusions

The presented results indicate the importance of choosing the proper type and amount of surfactant for nanoparticles synthesis. Surfactants influence not only the separation of the nanoparticles, but also their size, shape, and magnetic response to the external magnetic field. Particle growth shows dependence on the characteristic of the surfactant, as well as its amount in respect to core precursors. In general, amines ensure the growth of more evenly shaped particles in comparison to fatty acids. On the other hand, tertiary amines cause flower-like nanoparticle growth. Mössbauer spectra demonstrate that the higher surfactant concentration causes magnetite-like nanoparticle structure, while lower concentrations lead to magnetite-like nanoparticles. In field Mössbauer measurements show evident contribution from both the maghemite and magnetite part of the spectra. Detailed studies on physicochemical properties of magnetic nanoparticles can significantly contribute to the development of nanoparticles, which can be successfully applied in magnetic hyperthermia treatment. Therefore, the described data shows the important role of surfactants during the synthesis of iron oxide nanoparticles. 

## Figures and Tables

**Table 1:** Summary of types of used surfactants together with their abbreviated names.

Nanoparticles Name	Used Surfactant
Fe_3_O_4_-OA	Oleic acid
Fe_3_O_4_-LA	Lauric acid
Fe_3_O_4_-PA	Palmitic acid
Fe_3_O_4_-SA	Stearic acid
Fe_3_O_4_-CA	Caprylic acid
Fe_3_O_4_-OLA	Oleylamine
Fe_3_O_4_-TOA	Trioctylamine
Fe_3_O_4_-HA	Hexylamine
Fe_3_O_4_-DOA	Dioctylamine
Fe_3_O_4_-TEA	Triethylamine

**Table 2:** Summary of estimated grain sizes from TEM images and X-ray diffraction. Lattice constants and lattice strain values were determined from XRD.

Nanoparticle	Surfactant Concentration (mmol)	Size (TEM) ± 2 (nm)	Size ± 2 (nm)	Lattice Constant ± 0.02 (Å)	Strain × 10^−3^ ± 0.5
Fe_3_O_4_-OA	4	11	12	8.38	2.8
	8	12	11	8.39	3.4
	16	10	11	8.40	2.1
Fe_3_O_4_-LA	4	12	11	8.39	3.2
	8	12	12	8.39	2.8
	16	13	11	8.39	2.8
Fe_3_O_4_-PA	4	12	11	8.39	2.4
	8	11	12	8.36	4.5
	16	8	9	8.38	4.5
Fe_3_O_4_-SA	4	17	14	8.38	3.0
	8	15	13	8.38	4.6
	16	16	13	8.39	2.9
Fe_3_O_4_-CA	4	16	11	8.35	5.6
	8	15	12	8.36	4.8
	16	14	12	8.35	6.3
Fe_3_O_4_-TOA	4	22	15	8.36	5.1
	8	19	15	8.37	3.7
	16	23	14	8.37	3.6
Fe_3_O_4_-HA	4	13	12	8.39	1.9
	8	10	11	8.38	2.4
	16	6	7	8.38	3.8
Fe_3_O_4_-DOA	4	25	17	8.39	1.8
	8	11	12	8.39	1.9
	16	16	11	8.36	2.9
Fe_3_O_4_-OLA	4	10	10	8.39	2.8
	8	8	7	8.38	3.2
	16	6	6	8.39	4.6
Fe_3_O_4_-TEA	4	29	14	8.37	4.3
	8	28	13	8.36	5.5
	16	31	13	8.37	3.2
